# Resting metabolic rate of obese patients under very low calorie ketogenic diet

**DOI:** 10.1186/s12986-018-0249-z

**Published:** 2018-02-17

**Authors:** Diego Gomez-Arbelaez, Ana B. Crujeiras, Ana I. Castro, Miguel A. Martinez-Olmos, Ana Canton, Lucia Ordoñez-Mayan, Ignacio Sajoux, Cristobal Galban, Diego Bellido, Felipe F. Casanueva

**Affiliations:** 1 Division of Endocrinology, Department of Medicine, Molecular and Cellular Endocrinology Area, Complejo Hospitalario Universitario de Santiago (CHUS), Instituto de Investigación Sanitaria de Santiago (IDIS), Travesia da Choupana street s/n, 15706 Santiago de Compostela, La Coruña Spain; 2 Medical Department Pronokal, Pronokal Group, Barcelona, Spain; 3 Intensive Care Division, Complejo Hospitalario Universitario de Santiago (CHUS), Santiago de Compostela, Spain; 4 Division of Endocrinology, Complejo Hospitalario Universitario de Ferrol and Coruña University, Ferrol, Spain; 5 CIBER de Fisiopatologia de la Obesidad y Nutricion (CIBERobn), Instituto Salud Carlos III, Santiago de Compostela, Spain

**Keywords:** Ketogenic diet, Very low-energy diet, Pronokal method, Protein diet, Obesity, Metabolic adaptation, Energy expenditure, Indirect calorimetry, DXA, Multifrequency BIA

## Abstract

**Background:**

The resting metabolic rate (RMR) decrease, observed after an obesity reduction therapy is a determinant of a short-time weight regain. Thus, the objective of this study was to evaluate changes in RMR, and the associated hormonal alterations in obese patients with a very low-calorie ketogenic (VLCK)-diet induced severe body weight (BW) loss.

**Method:**

From 20 obese patients who lost 20.2 kg of BW after a 4-months VLCK-diet, blood samples and body composition analysis, determined by DXA and MF-Bioimpedance, and RMR by indirect calorimetry, were obtained on four subsequent visits: visit C-1, basal, initial fat mass (FM) and free fat mass (FFM); visit C-2, − 7.2 kg in FM, − 4.3 kg in FFM, maximal ketosis; visit C-3, − 14.4 kg FM, − 4.5 kg FFM, low ketosis; visit C-4, − 16.5 kg FM, − 3.8 kg FFM, no ketosis. Each subject acted as his own control.

**Results:**

Despite the large BW reduction, measured RMR varied from basal visit C-1 to visit C-2, − 1.0%; visit C-3, − 2.4% and visit C-4, − 8.0%, without statistical significance. No metabolic adaptation was observed. The absent reduction in RMR was not due to increased sympathetic tone, as thyroid hormones, catecholamines, and leptin were reduced at any visit from baseline. Under regression analysis FFM, adjusted by levels of ketonic bodies, was the only predictor of the RMR changes (R^2^ = 0.36; *p* < 0.001).

**Conclusion:**

The rapid and sustained weight and FM loss induced by VLCK-diet in obese subjects did not induce the expected reduction in RMR, probably due to the preservation of lean mass.

**Trial registration:**

This is a follow up study on a published clinical trial.

**Electronic supplementary material:**

The online version of this article (10.1186/s12986-018-0249-z) contains supplementary material, which is available to authorized users.

## Background

It is widely accepted that during periods of energy deficit or restriction (eg., weight-loss diets), the human body tends to diminish energy expenditure by increasing the efficiency in its use and by decreasing the resting metabolic rate (RMR) [18]. This phenomenon of metabolic adaptation to weight reduction is called adaptive thermogenesis, defined as a decrease in RMR out of proportion to the decrease in body mass [5, 12]. Various groups have observed this phenomenon during obesity treatments independently of the strategy employed, including diet, exercise, diet plus exercise, pharmacologic treatments, and surgical interventions, and suggest that metabolic adaptation predisposes weight reduced obese patients to weight regain.

Published research has shown that a very low calorie ketogenic (VLCK)-diet was able to induce a significant weight loss and maintained their efficacy along 2 years [10, 11]. Because VLCK-diets target body fat mass (FM) with little reduction in fat free mass (FFM) [6], the working hypothesis was that VLCK-diet may induce a minor or null reduction in the RMR, thus preventing body weight regain.

The main target of this study was to observe the changes in RMR induced by a VLCK-diet in obese subjects, as well as the hormonal and metabolic alterations associated with that change.

## Methods

### Study population

This is a follow up study on a published clinical trial [6]. It was an open, uncontrolled, nutritional intervention clinical trial conducted for 4 months, and performed in a single center.

The patients attending the Obesity Unit at the Complejo Hospitalario Universitario of Santiago de Compostela, Spain to receive treatment for obesity were consecutively invited to participate in this study.

The inclusion criteria were, age 18 to 65 years, body mass index (BMI) ≥30 kg/m^2^, stable body weight in the previous 3 months, desire to lose weight, and a history of failed dietary efforts. The main exclusion criteria were, diabetes mellitus, obesity induced by other endocrine disorders or by drugs, and participation in any active weight loss program in the previous 3 months. In addition, those patients with previous bariatric surgery, known or suspected abuse of narcotics or alcohol, severe depression or any other psychiatric disease, severe hepatic insufficiency, any type of renal insufficiency or gouts episodes, nephrolithiasis, neoplasia, previous events of cardiovascular or cerebrovascular disease, uncontrolled hypertension, orthostatic hypotension, and hydroelectrolytic or electrocardiographic alterations, were excluded. Females who were pregnant, breast-feeding, or intending to become pregnant, and those with child-bearing potential and not using adequate contraceptive methods, were also excluded. Apart from obesity and metabolic syndrome, participants were generally healthy individuals.

The study protocol was in accordance with the Declaration of Helsinki and was approved by the Ethics Committee for Clinical Research of Galicia, Santiago de Compostela, Spain (registry 2010/119). Participants gave informed consent before any intervention related to the study. Participants received no monetary incentive.

### Nutritional intervention

All the patients followed a VLCK diet according to a commercial weight loss program (PNK method®), which includes lifestyle and behavioral modification support. The intervention included an evaluation by the specialist physician conducting the study, an assessment by an expert dietician, and exercise recommendations. This method is based on a high-biological-value protein preparations obtained from cow milk, soya, avian eggs, green peas and cereals. Each protein preparation contained 15 g protein, 4 g carbohydrates, 3 g fat, and 50 mg docohexaenoic acid, and provided 90–100 kcal.

The weight loss program has five steps (Additional file 1: Figure S1) and adheres to the most recent guidelines of 2015 European Food Safety Authority (EFSA) on total carbohydrates intake [3]. The first three steps consist of a VLCK diet (600–800 kcal/day), low in carbohydrates (< 50 g daily from vegetables) and lipids (only 10 g of olive oil per day). The amount of high-biological-value proteins ranged between 0.8 and 1.2 g per each kg of ideal body weight, to ensure patients were meeting their minimal body requirements and to prevent the loss of lean mass. In step 1, the patients ate high-biological-value protein preparations five times a day, and vegetables with low glycemic indexes. In step 2, one of the protein servings was substituted by a natural protein (e.g., meat or fish) either at lunch or at dinner. In step 3, a second serving of low fat natural protein was substituted for the second serving of biological protein preparation. Throughout these ketogenic phases, supplements of vitamins and minerals supplements, such as K, Na, Mg, Ca, and omega-3 fatty acids, were provided in accordance to international recommendations [22]. These three steps were maintained until the patient lost the target amount of weight, ideally 80%. Hence, the ketogenic steps were variable in time depending on the individual and the weight loss target.

In steps 4 and 5, the ketogenic phases were ended by the physician in charge of the patient based on the amount of weight lost, and the patient started a low-calorie diet (800–1500 kcal/day). At this point, the patients underwent a progressive incorporation of different food groups and participated in a program of alimentary re-education to guarantee the long-term maintenance of the weight loss. The maintenance diet, consisted of an eating plan balanced in carbohydrates, protein, and fat. Depending on the individual the calories consumed ranged between 1500 and 2000 kcal/day, and the target was to maintain the weight lost and promote healthy life styles.

During this study, the patients followed the different steps of the method until they reach the target weight or up to a maximum of 4 months of follow-up, although patients remained under medical supervision for the following months.

### Schedule of visits

**Fig. 1:**
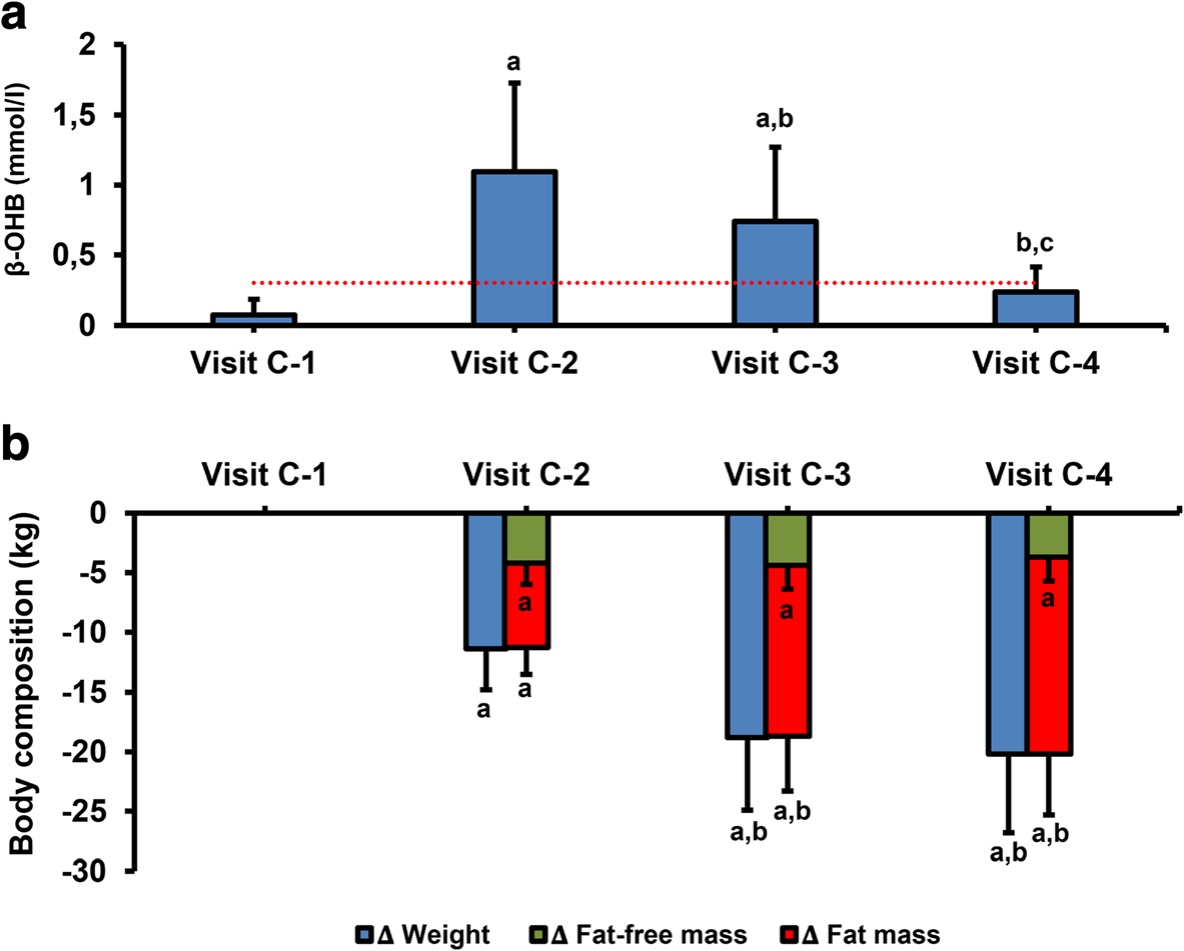
Ketone bodies (**a**) and body composition (**b**) during the study. The broken line represents the level at which the existence of ketosis is defined.^a^*P* < 0.05 compared with Visit C-1; ^b^*P* < 0.05 compared with Visit C-2; ^c^*P* < 0.05 compared with Visit C-3 (Repeated measures ANOVA with Tukey’s adjustment for multiple comparisons). β-OHB: β-hydroxy-butyrate

Throughout the study, the patients completed a maximum of 10 visits with the research team (every 15 ± 2 days), of which four were for a complete (C) physical, anthropometric and biochemical assessment, and the remaining visits were to control adherence and evaluation of potential side effects. The four complete visits were made according to the evolution of each patient through the steps of ketosis as follows: visit C-1 (baseline), normal level of ketone bodies; visit C-2, maximum ketosis (approximately 1–2 months of treatment); visit C-3, reduction of ketosis because of partial reintroduction of normal nutrition (2–3 months); visit C-4 at 4 months, no ketosis (Additional file 1: Figure S1 and Fig. 1a). The total ketosis state lasted for 60–90 days only. In all the visits, patients received dietary instructions, individual supportive counsel, and encouragement to exercise on a regular basis using a formal exercise program. Additionally, a program of telephone reinforcement calls was instituted, and a phone number was provided to all participants to address any concern.

### Anthropometric assessment

All anthropometric measurements were undertaken after an overnight fast (8 to 10 h), under resting conditions, in duplicate, and performed by well-trained health workers. Participant’s body weights were measured to the nearest 0.1 kg on the same calibrated electronic device (Seca 220 scale, Medical Resources, EPI Inc. OH, USA), in underwear and without shoes. BMI was calculated by dividing body weight in kilograms by the square of height in meters (BMI = weight (kg)/height^2^ (m).

### Resting metabolic rate

The RMR was measured by indirect calorimetry using a portable desktop metabolic system (FitMate PRO, Cosmed, Rome, Italy) and under overnight fasting conditions. Participants were instructed to arrive at the hospital by car, to minimize vigorous physical activity during the 24 h prior to the measurement, and to avoid drinking caffeinated beverages for at least 12 h before testing. All participants rested supine for at least 20 min. During this resting time, the body composition (bone mineral density, lean body mass and fat mass) was determined, and then rested in sitting position in a quiet and darkened room for a further 15 min before the test.

Test-re-test validation was performed and after resting, oxygen consumption was measured continuously for 15 min under thermo-neutral conditions, and the final 10 min of data were used to calculate RMR. The FitMate uses a turbine flow meter for measuring ventilation and a galvanic fuel cell oxygen sensor for determining the fraction of oxygen in expired gases. Moreover, it has sensors for the measurement of temperature, humidity, and barometric pressure for use in internal calculations. The FitMate uses standard metabolic formulas to estimate oxygen consumption, and RMR is calculated using a predetermined respiratory quotient (RQ) of 0.85. During the measurement period, participants remained sitting, breathed normally, and were instructed to remain awake, and to avoid talking, fidgeting and hyperventilating. The reliability of measuring RMR with Cosmed’s FitMate metabolic system have been determined in several previous studies [9, 15, 21], and by in house controls (Additional file 2: Figure S2).

For the purposes of this study measured RMR or the crude values provided by the method were obtained and expected-RMR was defined as the variation in energy expenditure that could be explained by the observed changes in fat-free mass (FFM), because FFM is the main determinant of RMR [13]. Firstly, we determined the basal energy equivalence per kilogram of FFM in our study population. Then, this quotient was multiplied by the amount of change in FFM between the baseline and each subsequent complete visit. Finally, this product was added to the basal measured RMR, and in this way the expected RMR for each complete visit was obtained.

This process is summarized by the following equation:

$$ \mathrm{RMR}\ \mathrm{expected}=\mathrm{RMR}\ {\mathrm{measured}}_{\mathrm{Baseline}}+\left[\left(\mathrm{RMR}\ {\mathrm{measured}}_{\mathrm{Baseline}}/{\mathrm{FFM}}_{\mathrm{Baseline}}\right)\ {\mathrm{X}\Delta \mathrm{FFM}}_{\mathrm{Visit}\hbox{-} \mathrm{Baseline}}\right]. $$

On the other hand, metabolic adaptation has been described as the change in RMR not explained by changes in FFM [8, 19], and is calculated as the difference between RMR measured at each complete visit and the expected RMR for that visit i.e., Metabolic adaptation = RMR measured – RMR expected.

### Total body composition

Body composition was first measured by dual-energy X-ray absorptiometry (DXA; GE Healthcare Lunar, Madison, USA).Daily quality control scans were acquired during the study period. No hardware or software changes were made during the course of the trial. Subjects were scanned using standard imaging and positioning protocols, while wearing only light clothing. For this study, the values of bone mineral density, lean body mass and FM that were directly measured by the GE Lunar Body Composition Software option. Some derivative values, such as bone mineral content, regional lean mass, FFM, and fat mass percentage (FM%), were also calculated.

### Multifrequency bioelectrical impedance

Multifrequency bioelectrical impedance (MF–BiA) was also used for determining body composition. FM, FM%, FFM, total body water, intra- and extracellular water, and skeletal muscle mass, were calculated with In Body 720 (In Body 720, Biospace Inc.,Tokyo, Japan). This technology is non-invasive and uses eight contact electrodes, which are positioned on the palm and thumb of each hand and on the front part of the feet and on the heels.

Multifrequency bioelectrical impedance uses the body’s electrical properties and the opposition to the flow of an electric current by different body tissues. The analyzer measures resistance at specific frequencies (1, 5, 50, 250, 500 and 1000 kHz) and reactance at specific frequencies (5, 50, and 250 kHz). The participants were examined lightly dressed, and the examination took less than 2 min and required only a standing position. The validity of this technology has been documented in previous studies [6].

### Determination of levels of ketone bodies

Ketosis was determined by measuring ketone bodies, specifically β-hydroxy-butyrate (β-OHB), in capillary blood by using a portable meter (GlucoMen LX Sensor, A. Menarini Diagnostics, Neuss, Germany). As with anthropometric assessments, all the determinations of capillary ketonemia were made after an overnight fast of 8 to 10 h. These measurements were performed daily by each patient during the entire VLCK diet, and the corresponding values were reviewed on the machine memory by the research team in order to control adherence. Additionally, β-OHB levels were determined at each visit by the physician in charge of the patient. The measurements reported as “low value” (< 0.2 mmol/l) by the meter were assumed as to be zero for the purposes of statistical analyses.

### Biochemical parameters

During the study all the patients were strictly monitored with a wide range of biochemical analyses. However, for the purposes of this work only certain values are reported. Serum tests for total proteins, albumin, prealbumin, retinol-binding protein, red cell and white cells counts, uric acid, urea, creatinine and urine urea were performed using an automated chemistry analyzer (Dimension EXL with LM Integrated Chemistry System, Siemens Medical Solutions Inc., USA).Thyroid-stimulating hormone (TSH), free thyroxine (FT4), and free triiodothyronine (FT3) were measured by chemiluminescence using ADVIA Centaur (Bayer Diagnostics, Tarrytown, NY, USA). All the biochemical parameters were measured at the 4 complete visits.

The overnight fasting plasma levels of leptin were measured using commercially available ELISA kits (Millipore, MA, USA). The fasting plasma levels of fractionated catecholamines (dopamine, adrenaline and noradrenaline) were tested by high pressure liquid chromatography (HPLC; Reference Laboratory, Barcelona, Spain).

### Statistical analysis

The data are presented as means (standard deviation). Each subject acted as his own control (baseline visit). The sample size of the current trial was calculated taking the weight loss after treatment (main variable) into account. It was calculated for an effect size ≥15 kg, and a α = 0.05, and a power (1-β) of 90%. Thus, the sample size was established at a minimum of 19 volunteers who finished the nutritional treatment. The sample size provided sufficient power to test for effects on a number of other metabolic variables of interest.

All statistical analyses were carried out using Stata statistical software, release 12.0 (Stata Corporation, College Station, TX, USA). A *p* < 0.05 was considered statistically significant. Changes in the different variables of interest from the baseline and throughout the study visits were analyzed following a repeated measures design. A repeated measures analysis of variance (ANOVA) test was used to evaluate differences between different measurement times, followed by post hoc analysis with Tukey’s adjustment for multiple comparisons. In addition, multivariate linear regression models were fitted to assess the potential predictive factors of RMR at each complete visit. The regression models included fat-free mass, FT3, catecholamines (i.e. noradrenaline, adrenaline and dopamine), leptin and β-OHB as plausible determinants of RMR.

## Results

Twenty obese patients, 12 females, age from 18 to 58 years (47.2 ± 10.2 yr) completed the study. Participants at baseline have a BMI of 35.5 ± 4.4 and body weight (BW) of 95.9 ± 16.3 kg, 45.6 ± 5.4% of which was fat. Other baseline characteristics and their corresponding changes during the study are presented in Tables 1 and 2, and have also been previously reported [6].

**Table 1:** Changes in anthropometry, energy expenditure and ketone bodies during the study

	VLCK diet			LC diet
	Visit C-1	Visit C-2	Visit C-3	Visit C-4
Diet time (days)		39.2 ± 8.4	89.7 ± 19.1	123.3 ± 17.6
Anthropometry				
Weight (kg)	95.9 ± 16.3	84.2 ± 13.0^a^	76.6 ± 11.1^a,b^	75.1 ± 11.8^a,b^
Weight change (kg)		−11.7 ± 3.7^d^	− 19.3 ± 6.4^d^	− 20.7 ± 6.9^d^
Weight change (%)		−12.0 ± 2.0^d^	− 19.7 ± 4.0^d^	− 21.3 ± 4.8^d^
Body mass index (kg/m^2^)	35.5 ± 4.4	31.2 ± 3.3^a^	28.4 ± 2.6^a,b^	27.8 ± 2.9^a,b^
Body fat _DXA_ (%)	45.6 ± 5.4	43.1 ± 6.1^a^	37.9 ± 5.8^a,b^	35.7 ± 5.9^a,b,c^
Fat mass _DXA_ (kg)	42.2 ± 9.1	35.0 ± 7.8^a^	27.8 ± 5.7^a,b^	25.7 ± 5.8^a,b^
Fat-free mass _DXA_ (kg)	52.8 ± 10.2	48.5 ± 9.2^a^	48.3 ± 9.1^a^	49.0 ± 9.7^a^
Muscle mass _MF-BIA_ (kg)	31.2 ± 6.6	29.3 ± 6.4^a^	29.4 ± 6.3^a^	29.6 ± 6.6^a^
Energy expenditure				
RMR-measured (kcal/d)	1978.1 ± 415.7	1913.1 ± 317.2^ns^	1860.3 ± 341.9^ns^	1776.6 ± 309.3^ns^
RMR-measured change (kcal/d)		−65.0 ± 343.7^ns^	− 117.8 ± 489.3^ns^	−201.5 ± 313.5^ns^
RMR-measured change (%)		−1.0 ± 18.8^ns^	−2.4 ± 25.6^ns^	−8.3 ± 15.0^ns^
RMR-expected _FFM-DXA_ (kcal/d)		1820.2 ± 379.9	1811.1 ± 369.4	1837.7 ± 389.3
Metabolic adaptation _FFM-DXA_ (kcal/d)		92.8 ± 339.5^ns^	49.1 ± 470.5^ns^	−61.0 ± 298.9^ns^
RMR/Fat-free mass _DXA_ (kcal/d/kg)	37.6 ± 5.5	40.1 ± 7.6^ns^	39.3 ± 8.7^ns^	36.5 ± 4.6^ns^
RMR-expected _MM-MF-BIA_ (kcal/d)		1856.1 ± 409.0	1863.1 ± 402.8	1873.6 ± 410.8
Metabolic adaptation _MM-MF-BIA_ (kcal/d)		56.9 ± 355.0^ns^	−2.8 ± 504.4^ns^	−96.9 ± 320.8^ns^
RMR/Muscle mass _MF-BIA_ (kcal/d/kg)	63.9 ± 10.5	66.9 ± 13.2^ns^	65.1 ± 15.2^ns^	60.9 ± 8.3^ns^
Ketone bodies				
β-OHB	0.0 ± 0.1	1.0 ± 0.6^a^	0.7 ± 0.5^a,b^	0.2 ± 0.1^b,c^

Data are presented as mean ± standard deviation. RMR: resting metabolic rate; FFM: fat-free mass; MM: muscle mass; β-OHB: β-hydroxy-butyrate

RMR-expected = RMR-measured _Baseline_ – ((RMR-measured _Baseline_/Fat-free mass _Baseline_) X Δ Fat-free mass _Visit-Baseline_)

Metabolic adaptation = RMR-measured – RMR-expected

^a^*P* < 0.05 compared with Visit C-1; ^b^*P* < 0.05 compared with Visit C-2; ^c^*P* < 0.05 compared with Visit C-3 (Repeated measures ANOVA with Tukey’s adjustment for multiple comparisons); ^d^*P* < 0.05 weight change significantly different from zero (Student’s *t*-test); ns: not significant

**Table 2:** Biochemical measurements during the study

	VLCK diet			LC diet
	Visit C-1	Visit C-2	Visit C-3	Visit C-4
Protein status				
Total proteins (g/dl)	7.2 ± 0.4	7.2 ± 0.4	7.1 ± 0.4	7.0 ± 0.4
Albumin (g/dl)	3.8 ± 0.2	4.1 ± 0.1^a^	3.9 ± 0.1^a,b^	3.8 ± 0.2^b^
Prealbumin (mg/dl)	26.6 ± 3.7	19.4 ± 3.2^a^	20.6 ± 3.5^a^	24.0 ± 3.6^a,b,c^
Retinol-binding protein (mg/dl)	4.7 ± 0.9	3.3 ± 0.7^a^	3.5 ± 0.7^a^	4.1 ± 0.7^a,b,c^
Lymphocytes (× 10^3^/μl)	1.9 ± 0.4	1.4 ± 0.5^a^	1.6 ± 0.5^a^	1.8 ± 0.5^b^
Renal function				
Serum uric acid (mg/dl)	5.1 ± 1.1	5.4 ± 0.9	5.4 ± 0.8	5.0 ± 0.8
Blood urea (mg/dl)	34.3 ± 10.0	26.3 ± 6.1^a^	34.1 ± 7.8^b^	33.1 ± 8.5^b^
Creatinine (mg/dl)	0.6 ± 0.1	0.6 ± 0.1	0.6 ± 0.1	0.6 ± 0.1
Glomerular filtration rate (ml/min/1,73 m^2^)	129.7 ± 65.3	128.4 ± 46.5	118.6 ± 35.6	110.2 ± 24.2
Nitrogen balance				
Urine urea (g/day)	27.4 ± 9.7	23.4 ± 5.2	31.3 ± 8.2^b^	33.2 ± 7.7^a,b^
Urine urea nitrogen (g/day)	12.8 ± 4.5	10.9 ± 2.4	14.6 ± 3.8^b^	15.5 ± 3.6^a,b^
Protein intake (g nitrogen/day)*	–	12	16.8	16.8
Nitrogen balance	–	1.0 ± 2.4	2.1 ± 3.8	1.2 ± 3.6

Data are presented as mean ± standard deviation.*Refers to the amount of proteins prescribed in each step of the diet. ^a^*P* < 0.05 compared with Visit C-1; ^b^*P* < 0.05 compared with Visit C-2; ^c^*P* < 0.05 compared with Visit C-3 (Repeated measures ANOVA with Tukey’s adjustment for multiple comparisons)

Although the patients underwent a total of 10 visits, the RMR and body composition analyses were synchronized with the ketone levels in four visits (Fig. 1a). Visit C-1 was the baseline visit, before starting the diet and with no ketosis (0.0 ± 0.1 mmol/L) and initial weight. Visit C-2 was at the time of maximum level of ketosis (1.0 ± 0.6 mmol/L) with 11.7 kg of BW loss. At visit C-3 (after 89.7 ± 19.1 days of VLCK), patients started the return to a normal diet and showed a reduction in ketone levels (0.7 ± 0.5 mmol/L) with 19.3 kg of BW loss. Finally, at visit C-4 the patients were out of ketosis (0.2 ± 0.1 mmol/L) with a total of 20.8 kg of weight lost (Table 1 and Fig. 1).

Most of the initial BW loss was in the form of fat mass (FM) with a minor reduction in fat free mass (FFA). The reduction in kg for FM and FFM respectively from baseline were; visit C-2 7.2 kg and 4.3 kg; visit C-3 14.4 kg and 4.5 kg; visit C-4 16.5 kg and 3.8 kg. (Table 1, Fig. 1b).

**Fig. 2:**
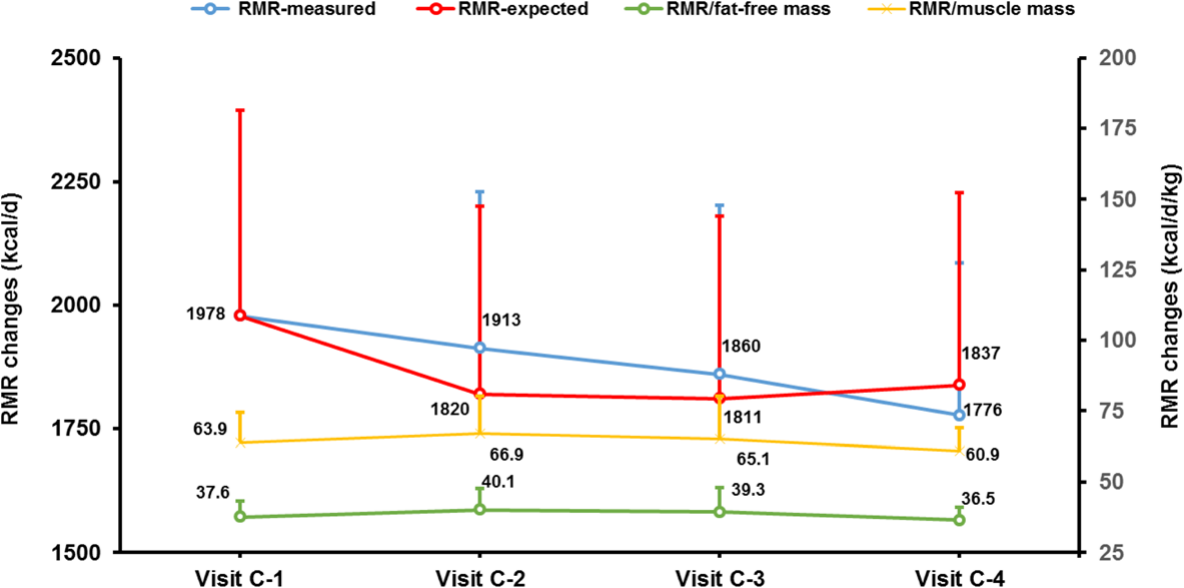
Resting metabolic rate (RMR) changes during the study. RMR-expected refers to the change in energy expenditure explained by changes in free fat mass (FFM) or muscle mass. Statistical analysis was performed by repeated measures ANOVA with Tukey’s adjustment for multiple comparisons)

The measured RMR was not significantly different from the baseline at any time during the study, although a downward trend in these values was observed (Fig. 2a). Compared to the baseline, at visits C-2, C-3 and C-4 the measured RMR varied − 1.0 ± 18.8%, − 2.4 ± 25.6% and − 8.3 ± 15.0%, respectively.

To investigate how much of the mild and non-significant decrease in RMR could be accounted for by FFM change, we used the baseline RMR data to generate an equation for calculating the expected-RMR in accordance with variations in FFM (Table 1). The difference between the measured and expected RMR defined the degree of metabolic adaptation. At visit C-2 (maximum ketosis), the measured RMR was 92.8 ± 339.5 kcal/d higher than expected RMR. At visit C-3, the measured RMR was 49.1 ± 470.5 kcal/d greater than expected RMR. Finally, at visit C-4, the measured RMR was 61.0 ± 298.9 kcal/d lower than the expected RMR. None of the differences between the measured and expected RMR was statistically different (Fig. 2 and Table 1), indicating that the phenomenon of metabolic adaptation was not present.

The observation that the VLCK-diet preserved the RMR in accordance to variations in FFM, thus avoiding the metabolic adaptation was reinforced by the maintenance of the RMR/FFM quotient during the study (Table 1 and Fig. 2). When, muscle mass evaluated by MF-BiA was employed in the analysis, instead of DXA, results on the expected and observed RMR were similar (Table 1 and Fig. 2).

The concern regarding the possible preservation of the RMR as a consequence of the presence of stressing factors induced by the VLCK-diet and the rapid weight loss was focused by a strict analysis of the protein metabolism. Although there were some differences in protein status, renal function and nitrogen balance-related parameters, none of them was considered as clinically relevant (Table 2). It is noteworthy that despite the considerable weight loss induced by the VLCK-diet, there was a positive nitrogen balance throughout the entire study. At visit C-2, the positive nitrogen balance was 1.0 ± 2.4, while at visits C-2 and C-3 it was 2.1 ± 3.8 and 1.2 ± 3.6, respectively. It was not possible to calculate the nitrogen balance at baseline since the protein intake was not assessed at that visit.

**Fig. 3:**
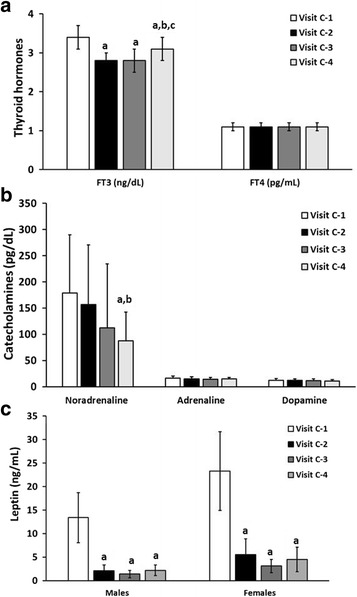
Thyroid hormones (**a**), Catecholamines (**b**) and Leptin (**c**) levels during the study. **a** Changes in Thyroid Hormones; **b**. Changes in Catecholamines; and **c**. Changes in Leptin. FT3: free triiodothyronine; FT4: tyroxine. ^a^*P* < 0.05 compared with Visit C-1; ^b^*P* < 0.05 compared with Visit C-2; ^c^*P* < 0.05 compared with Visit C-3 (Repeated measures ANOVA with Tukey’s adjustment for multiple comparisons)

Besides the FFM, that is considered the major contributing factor, several variables have been described as positive determinants of the RMR, including thyroid hormones, catecholamines, leptin and ketone bodies. In this study, the level of influence of these mentioned factors on the measured RMR was determined during the study. As Fig. 3 shows TSH and free T4 did not significantly change, free T3 had a significant although expected decrease at visit C-2, and thereafter. Adrenaline and dopamine did not significantly change during the study, but noradrenaline had a progressive decrease in their plasma levels that reached significant differences at visit C-4. Similarly, leptin values were severely reduced at visit 2, 3 and 4 in accordance with the FM reduction.

Linear regression models reveals that when adjusted by β-OHB, FFM was the best predictor of RMR (coefficient β = 20–31; *p* < 0.05, Table 3) explaining more than 40% of the variability of RMR (see Additional file 3: Table S1).

**Table 3:** Independent effects of fat-free mass and β-hydroxy-butyrate on resting metabolic rate at each visit

Regression equations		*Corrected R* ^2^	Coefficients B	95% CI	*P* Value
Visit C-1					
Fat-free mass		–	30.7	18.4–43.0	< 0.001
B-hydroxy-butyrate		–	− 791.7	− 1925.6 – 342.2	0.159
		0.64			< 0.001
Visit C-2					
Fat-free mass		–	20.0	6.0–34.0	0.008
β-hydroxy-butyrate		–	183.1	− 23.1 – 389.3	0.078
		0.38			0.015
Visit C-3					
Fat-free mass		–	14.8	−3.5 – 33.3	0.108
β-hydroxy-butyrate		–	146.2	− 172.4 – 464.9	0.346
		0.15			0.239
Visit C-4					
Fat-free mass		–	25.1	14.3–35.8	< 0.001
β-hydroxy-butyrate		–	9.9	− 586.5 – 606.3	0.972
		0.62			< 0.001

95% CI: 95% confidence interval. Multivariate regression analysis

## Discussion

To the best of our knowledge this study is the first assessing the effect of VLCK-diet on the RMR of obese patients. The main findings of this work were: 1) the rapid and sustained weight reduction induced by the VLCK-diet did not induce the expected drop in RMR, 2) this observation was not due to a sympathetic tone counteraction through the increase of either catecholamines, leptin or thyroid hormones, 3) the most plausible cause of the null reduction of RMR is the preservation of lean mass (muscle mass) observed with this type of diet.

The greatest challenge in obesity treatment is to avoid weight recovery sometime after the previous reduction. In fact, after one or few years the most obese patients recover or even increase their weight, previously reduced by either, dietetic, pharmacological or behavioral treatments [8], bariatric surgery being the only likely exception [7]. Since obesity reduction is accompanied by a slowing of energy expenditure in sedentary individuals, mostly RMR, this fact has been blamed for this negative outcome of the diet-based treatments [12]. RMR is recognized as the major component of total energy expenditure, being responsible for about 75% of daily total energy expenditure in Western societies [1, 16]. Therefore any RMR reduction after treatment, translates in a large impact on energy balance, making subjects more prone to weight regain over time [17]. This phenomenon was called metabolic adaptation or adaptive thermogenesis, indicating that RMR is reduced after weight loss, and furthermore that this reduction is usually larger than expected or out of proportion with the decrease in fat or fat free mass [2].Therefore, preservation of initial RMR after weight loss could play a critical role in facilitating further weight loss and preventing weight regain in the long-term [4].

We have observed that the obesity-reduction by a VLCK-diet (Pnk method ®) was maintained 1 and 2 years after its completion [10, 11]. Although that follow up was not long enough, the finding may be of particular importance for long-term effects. The present work shows that in a group of obese patients treated with a VLCK-diet, the RMR was relatively preserved, remaining within the expected limits for the variations in FFM, and avoided the metabolic adaptation phenomenon. Because FFM includes total body water, bone minerals and protein [14], the results were corroborated by analyzing the FFM without bone minerals and total body water (muscle mass).

As the mechanisms supporting the metabolic adaptation phenomenon are not known, unraveling the reasons behind the present findings is challenging enough in itself. Changes in any circulating hormone that participate in thermogenesis could be the explanation for the absence of a reduction in RMR, for example a concomitant increase in the sympathetic system activity, either directly or indirectly. An increase in thyroid hormones generated by the VLCK-diet was discarded because free T3 experienced the well described reduction after losing weight [20, 24] without alterations in free T4 or TSH. As thermogenesis in humans is largely a function of the sympathetic nervous system activity, and that activity decreases in response to weight loss the results here reported may be the net result of a maintenance or relative increase in the plasma catecholamine levels. However, it was found that adrenaline and dopamine remained unchanged throughout the study, while noradrenaline decreased considerably discarding their contribution to any increase in the activity of the autonomic nervous system. Leptin experienced a rapid decline in circulation in situations of weight reduction, although the reduction is observed in energy restriction states it occurs before any change in body weight [8]. On the other hand, leptin positively has been associated with sympathetic nervous system activity in humans, and weight loss associated changes in RMR and fat oxidation were previously related to leptin levels changes [25]. If leptin is sensitive to the energy flux and activate the autonomic nervous system, the absence of metabolic adaptation here observed could be due to a leptin increase, or maintenance in the basal levels. However, in this work, leptin levels decreased in accordance to the weight reduction.

Then, an expected increase in thyroid hormones, catecholamines, or leptin levels was discarded as explanation for the observed minor or absent reduction in RMR. This was also endorsed by the undertook multiple regression analysis (Table 3). In this analysis only the FFM (DXA) or the muscle mass (MF-BIA) appear as a plausible explanation for the maintenance of RMR activity. In fact, a clear preservation of FFM was reported in obese subjects on VLCK-diet, in whom 20 kg reduction after 4 months of treatment was accompanied by less than 1 kg of muscle mass lost [6]. The assumption of muscle mass preservation is also supported by the data on kidney function (Table 2) which shows that not only was renal activity not altered as reported in other studies [23] but that even the nitrogen balance was positive.

The strength of this study is its longitudinal design, which allows the evaluation of the time-course of changes of RMR during a VLCK diet, by comparing each subject to himself, as his own control. The scarce number of subjects and the short duration of this study might be a limitation, since one cannot make claims regarding the RMR status long-term after the completion of the VLCK diet. However, no significant variations in body weight had been observed after 4 months in previous studies [10, 11]. In addition, although participants were instructed to exercise on a regular basis using a formal exercise program, we could not verify adherence to this instruction which precludes determining whether changes in physical activity patterns affected study outcomes. In the current work a portable device that allows for easier measurement of RMR and with lower cost was employed. This approach may lead to errors when compared with the gold standard, Deltatrac, but it is an easy-to-use metabolic system for determining RMR and VO2 in clinical practice with a better accuracy than predictive eqs. [9]. The Deltatrac device is expensive and requires careful calibration. The Fitmate has been previously validated as a suitable alternative to the traditional indirect calorimetry by both in-house analysis (Additional file 1: Figure S1), as well as by previous studies. Despite not measuring CO2 production it is a very convenient in the clinical setting assuming a minimal error of analysis.

## Conclusions

In summary, this study shows that the treatment of obese patients with a VLCK-diet favors the maintenance of RMR within the expected range for FFM changes and avoids the metabolic adaptation phenomenon. This finding might explain the long-term positive effects of VLCK-diets on weight loss. Although, the mechanisms by which this effect could be justified are unclear, classical determinants of the energy expenditure, as thyroid hormones, catecholamines as well as leptin were discarded. The relative good preservation of FFM (muscle mass) observed with this dietetic approach could be the cause for the absence of metabolic adaptation.

## Additional files

Additional file 1: **Figure S1.** Nutritional intervention program and schedule of visits. Visit C-4 was performed at the end of the study according to each case, once the patient achieved the target weight or maximum at 4 months of follow-up. (PDF 390 kb)

Additional file 2: **Figure S2.** Bland Altman plots of Resting Metabolic Rate (RMR) for Cosmed’s Fitmate device compared to the Deltatrac. (PDF 11 kb)

Additional file 3: **Table S1.** Independent effects of fat-free mass, free triiodothyronine, catecholamines, leptin and β-hydroxy-butyrate on resting metabolic rate at each visit. (DOCX 32 kb)

## Abbreviations

BMI: Body mass index
BW: Body weight
DXA: Dual-energy X-ray absorptiometry
EFSA: European Food Safety Authority
FFM: Fat free mass
FM: Fat mass
MF-BIA: Multifrequency bioelectrical impedance
RMR: Resting metabolic rate
RQ: Respiratory quotient
VLCK: Very low calorie ketogenic diet

## References

[1] Black AE, Coward WA, Cole TJ, Prentice AM (1996). Human energy expenditure in affluent societies: an analysis of 574 doubly-labelled water measurements. Eur J Clin Nutr.

[2] Doucet E, St-Pierre S, Almeras N, Despres JP, Bouchard C, Tremblay A (2001). Evidence for the existence of adaptive thermogenesis during weight loss. Br J Nutr.

[3] EFSA Panel on Dietetic Products, Nutrition And Allergies (NDA) (2015). Scientific opinion on the essential composition of total diet replacements for weight control. EFSA J.

[4] Fothergill E, Guo J, Howard L, Kerns JC, Knuth ND, Brychta R, Chen KY, Skarulis MC, Walter M, Walter PJ, Hall KD (2016). Persistent metabolic adaptation 6 years after "the biggest loser" competition. Obesity (Silver Spring).

[5] Galgani JE, Santos JL (2016). Insights about weight loss-induced metabolic adaptation. Obesity (Silver Spring).

[6] Gomez-Arbelaez D, Bellido D, Castro AI, Ordonez-Mayan L, Carreira J, Galban C, Martinez-Olmos MA, Crujeiras AB, Sajoux I, Casanueva FF (2017). Body composition changes after very low-calorie-ketogenic diet in obesity evaluated by three standardized methods. J Clin Endocrinol Metab..

[7] Inge TH, Courcoulas AP, Jenkins TM, Michalsky MP, Helmrath MA, Brandt ML, Harmon CM, Zeller MH, Chen MK, Xanthakos SA, Horlick M, Buncher CR, Teen LC (2016). Weight loss and health status 3 years after bariatric surgery in adolescents. N Engl J Med.

[8] Knuth ND, Johannsen DL, Tamboli RA, Marks-Shulman PA, Huizenga R, Chen KY, Abumrad NN, Ravussin E, Hall KD (2014). Metabolic adaptation following massive weight loss is related to the degree of energy imbalance and changes in circulating leptin. Obesity (Silver Spring).

[9] Lupinsky L, Singer P, Theilla M, Grinev M, Hirsh R, Lev S, Kagan I, Attal-Singer J (2015). Comparison between two metabolic monitors in the measurement of resting energy expenditure and oxygen consumption in diabetic and non-diabetic ambulatory and hospitalized patients. Nutrition.

[10] Moreno B, Bellido D, Sajoux I, Goday A, Saavedra D, Crujeiras AB, Casanueva FF (2014). Comparison of a very low-calorie-ketogenic diet with a standard low-calorie diet in the treatment of obesity. Endocrine.

[11] Moreno B, Crujeiras AB, Bellido D, Sajoux I, Casanueva FF (2016). Obesity treatment by very low-calorie-ketogenic diet at two years: reduction in visceral fat and on the burden of disease. Endocrine.

[12] Muller MJ, Bosy-Westphal A (2013). Adaptive thermogenesis with weight loss in humans. Obesity (Silver Spring).

[13] Muller MJ, Bosy-Westphal A, Kutzner D, Heller M (2002). Metabolically active components of fat-free mass and resting energy expenditure in humans: recent lessons from imaging technologies. Obes Rev.

[14] Muller MJ, Braun W, Pourhassan M, Geisler C, Bosy-Westphal A (2016). Application of standards and models in body composition analysis. Proc Nutr Soc.

[15] Nieman DC, Austin MD, Benezra L, Pearce S, McInnis T, Unick J, Gross SJ (2006). Validation of Cosmed's FitMate in measuring oxygen consumption and estimating resting metabolic rate. Res Sports Med.

[16] Ravussin E, Lillioja S, Anderson TE, Christin L, Bogardus C (1986). Determinants of 24-hour energy expenditure in man. Methods and results using a respiratory chamber. J Clin Invest.

[17] Ravussin E, Lillioja S, Knowler WC, Christin L, Freymond D, Abbott WG, Boyce V, Howard BV, Bogardus C (1988). Reduced rate of energy expenditure as a risk factor for body-weight gain. N Engl J Med.

[18] Rosenbaum M, Hirsch J, Gallagher DA, Leibel RL (2008). Long-term persistence of adaptive thermogenesis in subjects who have maintained a reduced body weight. Am J Clin Nutr.

[19] Rosenbaum M, Leibel RL (2010). Adaptive thermogenesis in humans. Int J Obes.

[20] Sjostrom L, Narbro K, Sjostrom CD, Karason K, Larsson B, Wedel H, Lystig T, Sullivan M, Bouchard C, Carlsson B, Bengtsson C, Dahlgren S, Gummesson A, Jacobson P, Karlsson J, Lindroos AK, Lonroth H, Naslund I, Olbers T, Stenlof K, Torgerson J, Agren G, Carlsson LM, Swedish Obese Subjects S (2007). Effects of bariatric surgery on mortality in Swedish obese subjects. N Engl J Med.

[21] Stewart CL, Goody CM, Branson R (2005). Comparison of two systems of measuring energy expenditure. JPEN J Parenter Enteral Nutr.

[22] SCOOP-VLCD T. Reports on tasks for scientific cooperation. Collection of data on products intendend for use in very-low-calorie-diets. Report Brussels European Comission 2002.

[23] Tagliabue A, Bertoli S, Trentani C, Borrelli P, Veggiotti P (2012). Effects of the ketogenic diet on nutritional status, resting energy expenditure, and substrate oxidation in patients with medically refractory epilepsy: a 6-month prospective observational study. Clin Nutr.

[24] Van Gaal LF, Maggioni AP (2014). Overweight, obesity, and outcomes: fat mass and beyond. Lancet.

[25] Westerterp-Plantenga MS, Nieuwenhuizen A, Tome D, Soenen S, Westerterp KR (2009). Dietary protein, weight loss, and weight maintenance. Annu Rev Nutr.

