# Supplementary figures and images for: Resting metabolic rate of obese patients under very low calorie ketogenic diet
Source: Nutr Metab (Lond). 2018 Feb 17;15:18. doi: 10.1186/s12986-018-0249-z (PMC5816424; doi:10.1186/s12986-018-0249-z)

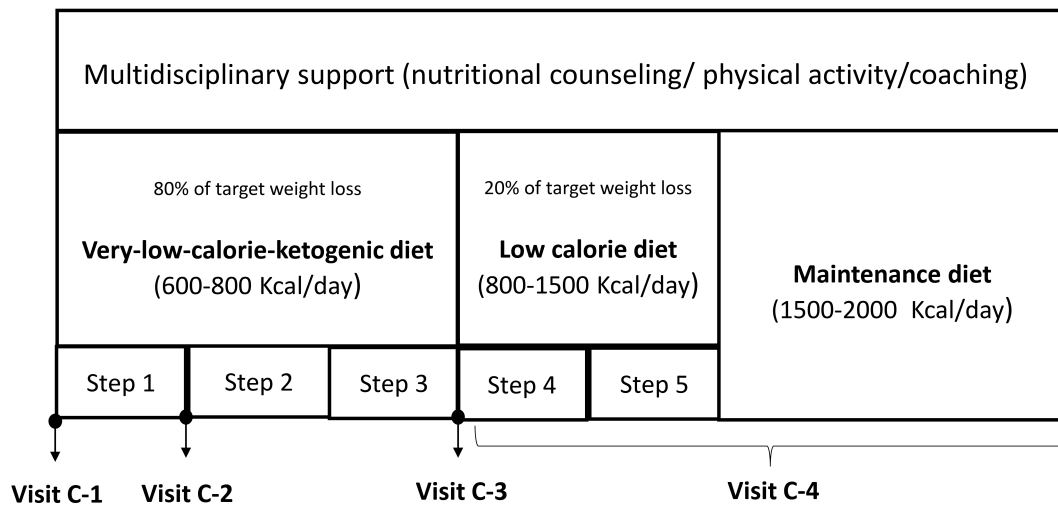

Figure 1S

Supplement: Supplementary file 1 — Figure S1. Nutritional intervention program and schedule of visits. Visit C-4 was performed at the end of the study according to each case, once the patient achieved the target weight or maximum at 4 months of follow-up. (PDF 390 kb) [file 12986_2018_249_MOESM1_ESM.pdf]

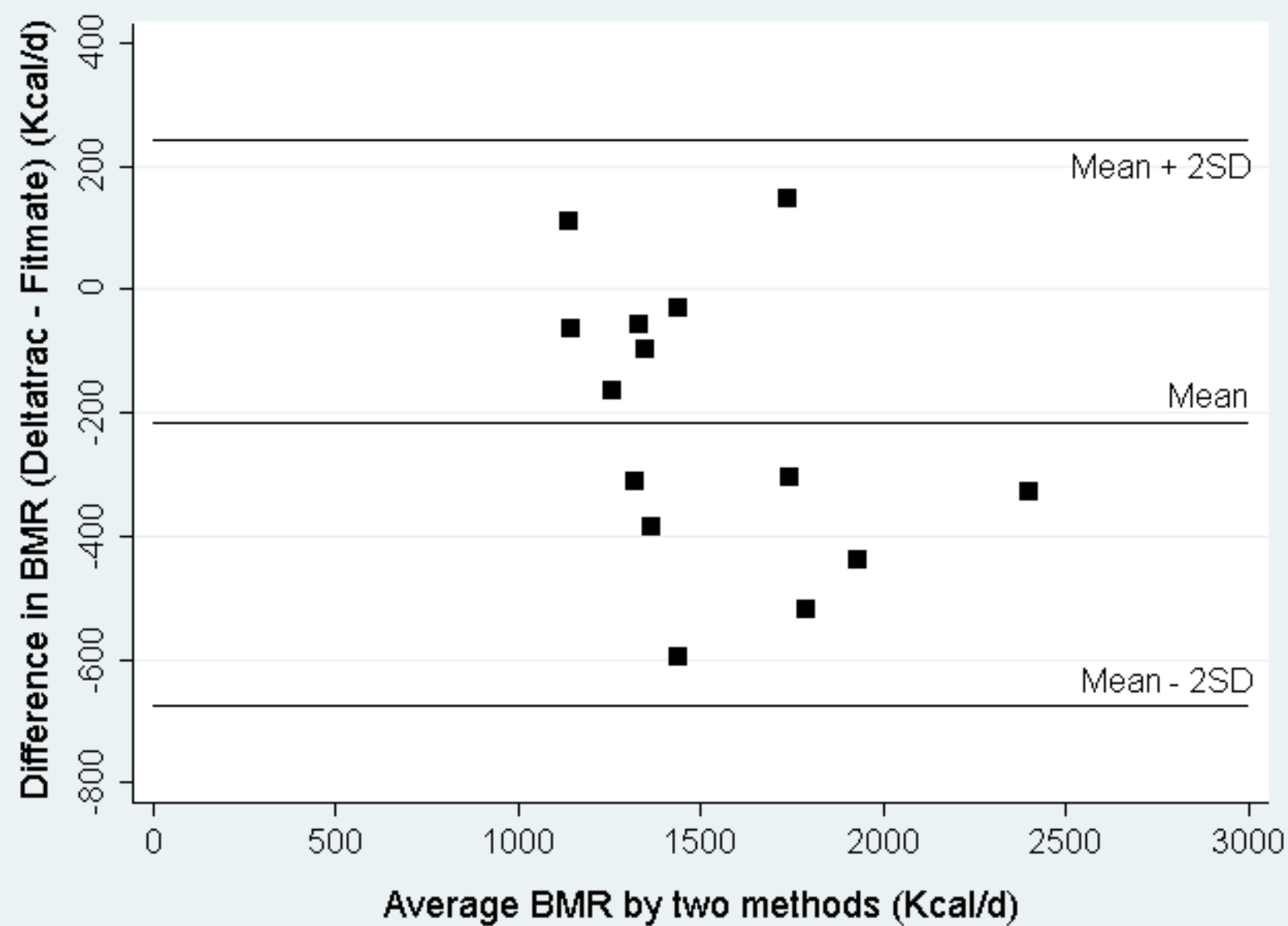

Supplement: Supplementary file 2 — Figure S2. Bland Altman plots of Resting Metabolic Rate (RMR) for Cosmed’s Fitmate device compared to the Deltatrac. (PDF 11 kb) [file 12986_2018_249_MOESM2_ESM.pdf]
