# Supplementary material for: Resting metabolic rate of obese patients under very low calorie ketogenic diet
Source: Nutr Metab (Lond). 2018 Feb 17;15:18. doi: 10.1186/s12986-018-0249-z (PMC5816424; doi:10.1186/s12986-018-0249-z)
Supplement: Supplementary file 3 — Table S1. Independent effects of fat-free mass, free triiodothyronine, catecholamines, leptin and β-hydroxy-butyrate on resting metabolic rate at each visit. (DOCX 32 kb) [file 12986_2018_249_MOESM3_ESM.docx]

**Additional file Table S1.** Independent effects of fat-free mass, free triiodothyronine, catecholamines, leptin and β-hydroxy-butyrate on resting metabolic rate at each visit.

|  | Regression equations | *Corrected R*^2^ | Coefficients B | 95%CI | *P* Value |
| --- | --- | --- | --- | --- | --- |
| Visit C-1 |  |  |  |  |  |
|  | Fat-free mass | --- | 32.2 | 6.3 – 58.0 | 0.020 |
|  | Fat mass | --- | -21.4 | -52.6 – 9.7 | 0.154 |
|  | Free triiodothyronine | --- | 277.1 | -267.6 – 821.9 | 0.280 |
|  | Noradrenaline | --- | 1.2 | -1.3 – 3.9 | 0.301 |
|  | Adrenaline | --- | -7.4 | -74.3 – 59.4 | 0.807 |
|  | Dopamine | --- | 62.9 | -55.8 – 181.6 | 0.261 |
|  | Leptin | --- | -2.5 | -30.4 – 25.3 | 0.841 |
|  | β-hydroxy-butyrate | --- | -1294.1 | -2733.5 – 145.3 | 0.072 |
|  |  | **0.76** |  |  | **0.036** |
| Visit C-2 |  |  |  |  |  |
|  | Fat-free mass | --- | 17.7 | -9.3 – 44.8 | 0.175 |
|  | Fat mass | --- | -2.6 | -46.2 – 40.9 | 0.896 |
|  | Free triiodothyronine | --- | 95.3 | -633.9 – 824.5 | 0.777 |
|  | Noradrenaline | --- | -0.1 | -1.8 – 1.5 | 0.840 |
|  | Adrenaline | --- | 11.4 | -47.9 – 70.8 | 0.676 |
|  | Dopamine | --- | -18.6 | -83.0 – 45.8 | 0.534 |
|  | Leptin | --- | -13.6 | -134.9 – 107.7 | 0.808 |
|  | β-hydroxy-butyrate | --- | 184.5 | -161.3 – 530.4 | 0.262 |
|  |  | **0.43** |  |  | **0.503** |
| Visit C-3 |  |  |  |  |  |
|  | Fat-free mass | --- | 47.9 | 18.7 – 77.1 | 0.006 |
|  | Fat mass | --- | 36.2 | -4.2 – 76.8 | 0.072 |
|  | Free triiodothyronine | --- | -910.7 | -1700.3 – -121.1 | 0.029 |
|  | Noradrenaline | --- | 0.3 | -0.7 – 1.5 | 0.454 |
|  | Adrenaline | --- | -34.5 | -92.9 – 23.7 | 0.204 |
|  | Dopamine | --- | 8.8 | -47.1 – 64.8 | 0.720 |
|  | Leptin | --- | 161.5 | 8.0 – 315.1 | 0.042 |
|  | β-hydroxy-butyrate | --- | 86.8 | -280.2 – 453.9 | 0.593 |
|  |  | **0.85** |  |  | **0.021** |
| Visit C-4 |  |  |  |  |  |
|  | Fat-free mass | --- | 25.8 | 5.8 – 45.8 | 0.016 |
|  | Fat mass | --- | 14.6 | -15.4 – 44.8 | 0.304 |
|  | Free triiodothyronine | --- | -209.4 | -785.1 – 366.2 | 0.437 |
|  | Noradrenaline | --- | 0.3 | -2.4 – 3.1 | 0.797 |
|  | Adrenaline | --- | 12.3 | -43.0 – 67.7 | 0.630 |
|  | Dopamine | --- | 3.2 | -55.1 – 61.6 | 0.903 |
|  | Leptin | --- | -9.6 | -105.6 – 86.4 | 0.828 |
|  | β-hydroxy-butyrate | --- | -28.5 | -935.5 – 878.3 | 0.945 |
|  |  | **0.66** |  |  | **0.084** |

95%CI: 95% confidence interval. Multivariate regression analysis.
